# Spinal manipulation and mobilisation in paediatrics – an international evidence-based position statement for physiotherapists

**DOI:** 10.1080/10669817.2024.2332026

**Published:** 2024-06-10

**Authors:** Anita R. Gross, Kenneth A. Olson, Jan Pool, Annalie Basson, Derek Clewley, Jenifer L. Dice, Nikki Milne

**Affiliations:** a Rehabilitation Sciences, McMaster University, Hamilton, Canada; b Northern Rehab Physical Therapy Specialists, DeKalb, Illinois, USA; c HU University of Applied Sciences, Epidemiologist, Emeritus Senior Researcher, Utrecht, The Netherlands; d Rehabilitation Sciences, University of Witwatersrand, Johannesburg, South Africa; e Doctor of Physical Therapy Division, Duke University, Durham, USA; f School of Physical Therapy, Texas Woman’s University, Houston, TX, USA; g THINK Paediatrics Research Group, Department of Physiotherapy, Faculty of Health Sciences and Medicine, Bond University, Gold Coast, Queensland, Australia; h International Organisation of Physiotherapists in Paediatrics (IOPTP)

**Keywords:** Spine, manipulation, mobilisation, infant, child, adolescent

## Abstract

**Introduction:**

An international taskforce of clinician-scientists was formed by specialty groups of World Physiotherapy – International Federation of Orthopaedic Manipulative Physical Therapists (IFOMPT) & International Organisation of Physiotherapists in Paediatrics (IOPTP) – to develop evidence-based practice position statements directing physiotherapists clinical reasoning for the safe and effective use of spinal manipulation and mobilisation for paediatric populations (<18 years) with varied musculoskeletal or non-musculoskeletal conditions.

**Method:**

A three-stage guideline process using validated methodology was completed: 1. Literature review stage (one scoping review, two reviews exploring psychometric properties); 2. Delphi stage (one 3-Round expert Delphi survey); and 3. Refinement stage (evidence-to-decision summative analysis, position statement development, evidence gap map analyses, and multilayer review processes).

**Results:**

Evidence-based practice position statements were developed to guide the appropriate use of spinal manipulation and mobilisation for paediatric populations. All were predicated on clinicians using biopsychosocial clinical reasoning to determine when the intervention is appropriate.

1. It is not recommended to perform:

• Spinal manipulation and mobilisation on infants.

• Cervical and lumbar spine manipulation on children.

•Spinal manipulation and mobilisation on infants, children, and adolescents for non-musculoskeletal paediatric conditions including asthma, attention deficit hyperactivity disorder, autism spectrum disorder, breastfeeding difficulties, cerebral palsy, infantile colic, nocturnal enuresis, and otitis media.

2. It may be appropriate to treat musculoskeletal conditions including spinal mobility impairments associated with neck-back pain and neck pain with headache utilising:

• Spinal mobilisation and manipulation on adolescents;

• Spinal mobilisation on children; or

• Thoracic manipulation on children for neck-back pain only.

3. No high certainty evidence to recommend these interventions was available.

Reports of mild to severe harms exist; however, risk rates could not be determined.

**Conclusion:**

Specific directives to guide physiotherapists’ clinical reasoning on the appropriate use of spinal manipulation or mobilisation were identified. Future research should focus on trials for priority conditions (neck-back pain) in children and adolescents, psychometric properties of key outcome measures, knowledge translation, and harms.

## Introduction

Safety concerns and uncertainty regarding the use of spinal manipulation and mobilisation to treat both non-musculoskeletal and musculoskeletal conditions in paediatric populations have been recognised as a priority problem based on political and policy decisions by multiple sources [1–6]. The escalation of the controversy regarding the safety and efficacy of spinal manipulation in paediatric populations sparked the need for the international physiotherapy community to develop position statements that utilise guideline development processes [6]. In August 2018, a social media video of an Australian Melbourne-based chiropractor manipulating the neck of a 2-week old baby triggered international media attention to the issue (Social Media Link) [1]. In 2019, Safer Care Victoria was commissioned to develop an independent review to identify evidence for both the safety and efficacy of spinal manipulation in children under 12 years of age. This resulted in recommendations to the Council of Australian Government [6,7] including that spinal manipulation should not be provided to children under 12 years of age for general wellness or to manage non-musculoskeletal conditions and prompted the Chiropractic Board of Australia to enforce an interim policy prohibiting the use of chiropractic spinal manipulation in children under the age of two years [3]. The Australian Physiotherapy Association echoed the position of the Safer Care Victoria report [8] and reiterated the need for an international evidence-based position statement for physiotherapists. An international physiotherapy taskforce of clinician-scientists was commissioned to develop an evidence-based practice position statement on the benefits and harms of spinal manipulation and mobilisation to treat paediatric populations (<18 years) by World Physiotherapy specialty groups: International Federation of Manipulative Physical Therapy (IFOMPT) and International Organisation of Physiotherapists in Paediatrics (IOPTP).

Evidence-based practice is a process of integrating the best available research evidence with clinical experience, the client’s values and circumstances, and the practice context [9,10]. The intent of the evidence-based practice position statement was to inform governments, payers, regulators, educators, clinicians, and clients to consider the evidence when developing care pathways, policies, and making decisions about the use and reimbursement of spinal manipulation and mobilisation for diverse conditions in paediatric populations. The taskforce adopted the following IFOMPT definitions [11]:

*Manipulation* - ‘A passive, high velocity, low amplitude thrust applied to a joint complex within its anatomical limit with the intent to restore optimal motion, function, and/or to reduce pain’.

*Mobilisation* - ‘A manual therapy technique comprising a continuum of skilled passive movements that are applied at varying speeds and amplitudes to joints, muscles or nerves with the intent to restore optimal motion, function, and/or to reduce pain’.

Both benefits (desirable effects) and harms (undesirable effects) are analysed in the development of the evidence-based practice position statements. Benefits are determined by evidence of favourable clinical outcomes. Harms, both direct and indirect, include adverse events that can range from mild symptoms to severe life-threatening events [12,13]. In order to judge the clinical outcomes of a treatment approach, the psychometric properties of the clinical outcome assessments used to measure the clinical outcomes must be understood [14]. Desirable effects must outweigh the undesirable effects to be applicable to clinical practice.

Our primary aim was twofold:

1. To systematically synthesise the research evidence and clinical expert opinion on benefits and harms of using spinal manipulation and mobilisation in paediatric populations for managing various conditions and associated impairments; and
2. To make specific evidence-based practice position statements on the appropriateness of their use.

## Methods

**Figure 1.**
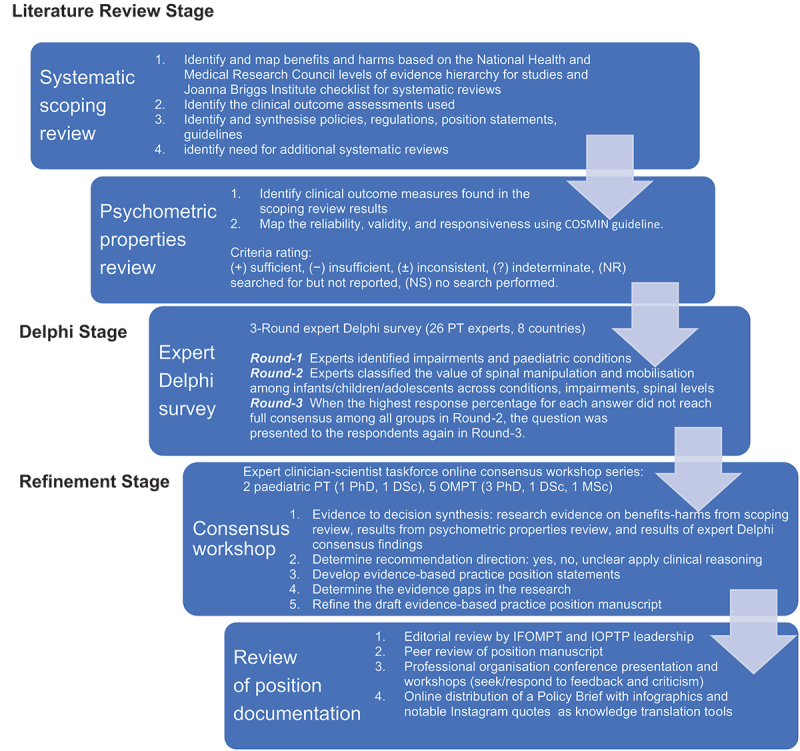
Stages of the guideline development process to formulate the position statements.

We developed the evidence-based practice position statement by using a three-stage guideline process as outlined in Figure 1: 1. Literature review stage, 2. Delphi stage, and 3. Refinement stage. Methods were adapted from health research methods for guideline development and the evidence-to-decision framework [15,16].

### Literature review stage

The literature review stage included one systematic scoping review on benefits and harms of spinal manipulation and mobilisation in infants (<2 years), children (2 to 12 years), and adolescents (13 to <18 years) [17]; and two systematic reviews [18,19] on the psychometric properties of the clinical outcome assessments used in studies included in the scoping review. A detailed protocol of each review was prospectively registered: 1) scoping review (https://osf.io/zm8e6) and 2) review of psychometric properties (https://osf.io/rn4ux/). All reviews have been published [17–19]. The scope of these documents were spinal manipulation and mobilisation for conditions in paediatric populations; adult populations and other interventions were excluded.

The level of evidence statement from the scoping review considered quality and quantity of evidence findings from systematic reviews and high-quality studies (≥5/7 on critical appraisal on the Mixed Methods Appraisal Tool) [20]. The resulting evidence statement was identified as very strong, strong, moderate, or limited for a positive favourable effect, negative unfavourable effect, or no significant effect. When results were mixed and further research may be warranted, the evidence was deemed ‘inconclusive’. Alternatively, when there was insufficient high-quality data from the reviews and studies, then ‘insufficient’ evidence was documented for the level of evidence statement. Evidence was based on the National Health and Medical Research Council levels of evidence hierarchy for studies [21], and Joanna Briggs Institute levels for systematic reviews [22]. Adverse events were classified according to the National Cancer Institute as mild – symptoms requiring self-care only; moderate – symptoms limiting activities of daily living or requiring medical care; and severe – medically significant symptoms resulting in life-threatening outcomes including urgent care, hospitalisation, or death [12].

The focus of the next two systematic reviews was on patient-reported, observer-reported, clinician-reported, and performance-based clinical outcome assessments for paediatric conditions identified in the scoping review [18,19]. The COSMIN criteria were used in the assessment of the measurement properties [14,23]. Psychometric property criteria were flagged as sufficient (+), insufficient (-), inconsistent (±), indeterminate (?), or searched for but not reported (NR) and the certainty of evidence was identified as high, moderate, low, or very low using a modified GRADE scale [14,23]. The evidence identified from these three reviews was used as the research foundation in the refinement stage.

### Delphi stage

A 3-Round Delphi survey of international physiotherapists on the clinical appropriateness of spinal manipulation and mobilisation for paediatric conditions and impairments was conducted [24]. The Delphi survey protocol had ethical clearance by Texas Tech Institutional Review Board (#L21–151) and Bond University (#NM03322). Physiotherapists from seven countries and five continents with paediatric or orthopedic manipulative therapy expertise identified by the IFOMPT and IOPTP member organisations were invited to participate. During the Delphi study, Round-1 identified impairments and conditions through open-ended questions while Round-2 and Round-3 established consensus. A Likert scale was used to rate the questions, with a threshold of seventy-five percent agreement on what was considered appropriate and not appropriate. The panelists received feedback from former rounds about their ratings between rounds. The expert insights and recommendations from the Delphi stage informed the refinement stage.

### Refinement stage

The refinement stage consisted of a two-phase process: 1. Consensus workshops; 2. Internal and Peer Review (see Figure 1). The use of reporting guidelines and the evidence-to-decision framework ensured that important criteria were considered and to inform the best available research decisions (See Box 1) [15,16]. The international physiotherapy taskforce of clinician-scientists including seven physiotherapists from five countries with expertise in research design, epidemiology, and paediatrics/orthopedic manual-manipulative physiotherapy clinical practice was appointed by World Physiotherapy specialty groups, IFOMPT and IOPTP. The physiotherapy taskforce of clinician-scientists conducted a series of online workshops to develop consensus and draw conclusions based on the summative analysis of the research evidence on benefits-harms from the scoping review, results from the psychometric properties reviews, and consensus findings from the expert Delphi panel using the decision rules outlined in Table 1. Summary of findings tables were generated compiling the research evidence (benefits, harms, psychometrics) and expert Delphi consensus findings by musculoskeletal and non-musculoskeletal conditions. The judgments made by the taskforce for each condition, the research evidence, and additional considerations used to inform each judgment were recorded. The beneficial or non-beneficial effects, the balance between benefits and harms, the certainty of the evidence, the Delphi expert recommendation findings, as well as acceptability and feasibility to the client and their carers were each considered in drawing judgments (See Box 1). The taskforce completed an iterative review, debate, and group consensus decision-making process with application of the decision rules to formulate clinical recommendations of appropriateness for spinal manipulation and mobilisation for each spinal region and paediatric population to treat the 14 conditions identified in the scoping review [17]. Themes were identified, collated, clustered, and summarised to develop the evidence-based practice position statements. These conclusions also encompassed relevant considerations about subgroups (i.e. by spinal region, by age, by manipulation or mobilisation).

**Box 1.** Criteria for evidence to decision framework adapted from Table 1 in Alonso-Coello and colleagues [16].

Benefits and Harms	How substantial are the desirable anticipated effects? How substantial are the undesirable anticipated effects?
Level of evidence^¶^	What is the overall level of evidence^¶^ of effect from the scoping review? What is the expert Delphi panels perspectives when the evidence was inconclusive or indeterminate? What are additional considerations for each subgroup (i.e., by spinal region, by age, by spinal manipulation or mobilisation), implementation, monitoring, evaluation, and research priorities?
Clinical outcome assessments of importance	What is the overall certainty of evidence^§^ for clinical outcome assessment used?
Balance	Does the balance between the desirable and undesirable effect favor the intervention?
Acceptability	Is the intervention acceptable to clients, carers, and healthcare providers? Is the intervention acceptable to key stakeholders?
Feasibility	Is the intervention feasible for clients, carers, and healthcare providers?

Key: ^¶^Level of Evidence based on Joanna Briggs Institute-Checklist for Systematic Reviews and Research Synthesis [22]; ^§^Certainty of Evidence using GRADE approach for systematic reviews as applied by COSMIN guidelines [23].

Identification of evidence-based gaps to establish the need for monitoring, setting priorities, and implementation for further research was conducted by the taskforce and assessed during the synthesis and summative analysis at the consensus workshops. Evidence gap maps; a visual matrix to represent the gaps in the current literature associated with our research question, were developed for each condition by age group. They provide a visual overview of areas with few or no studies and areas with sufficient primary studies for evidence synthesis [25,26]. Further research was recommended when the recommendation for clinical use was ‘Yes’ or ‘Unclear’. No research was recommended when the taskforce’s recommendation was ’No’, ‘not recommended for clinical use’.

Refinement of the position statements also occurred as the taskforce presented their evidence-to-decision processes and resultant evidence-based practice position statements at international professional association presentations and workshops, editorial review and publication, online distribution of a policy brief with infographics and notable Instagram quotes for knowledge translation implementation, and review from the leadership of IFOMPT and IOPTP. Infographics were designed for the reviews, Delphi study, and position statements to ensure easy access and knowledge translation for clients, clinicians and policy makers to the relevant findings and position statements. The dissemination and response to feedback of the position statements, policy brief, infographics, notable quotes occurred in concert with publication to refine the message delivery and knowledge translation.

**Table 1.** Decision rules for final recommendations and directives are listed. They were based on summative analysis of one scoping review, two reviews of psychometric properties, and one 3-round expert Delphi panel survey.

Directive	Decision Rule
NO	When the scoping review summary statement was insufficient, inconclusive, no data, or evidence of no effect, and the Delphi study consensus was either not recommended 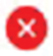 or the condition was not identified by the Delphi expert panel as an appropriate condition to treat with the intervention, the taskforce recommendation is ‘No’ indicating the intervention is not recommended for clinical use. The taskforce may change an ‘Unclear’ to a recommendation of ‘No’, indicating the intervention is not recommended for clinical use, due to evidence of safety concerns or a lack of biomechanical and neurophysiological plausibility in application of spinal manipulation or mobilisation for a specific paediatric condition or impairment.
UNCLEAR	When the scoping review summary statement concluded insufficient, inconclusive, no data, or evidence of no effect, and the Delphi study consensus was positive 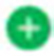 or did not reach consensus 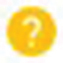 for clinical use, the taskforce clinical recommendation is ‘Unclear’ indicating that the clinician must use appropriate biopsychosocial clinical reasoning to determine if the intervention is appropriate. When the scoping review summary statement was inconclusive for clinical use and the Delphi study did not reach consensus 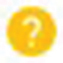 for or against clinical use, the taskforce recommendation is also ‘Unclear’ indicating that the clinician must use biopsychosocial clinical reasoning to determine when the intervention is appropriate.
YES	When the scoping review summary statement was a conclusive ‘Yes’ in support of the intervention, the psychometric properties were reliable, valid, and responsive to change a well as the Delphi consensus was positive 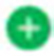 for clinical use, the taskforce clinical use recommendation is ‘Yes’ for the intervention to be recommended.

## Results

We have based the resulting seven (7) position statements on three systematic reviews (1 systematic scoping review, 2 reviews of psychometric properties), one expert Delphi survey, and a summative analysis by a taskforce of clinician-scientists. The summary of findings tables (Table 2 by musculoskeletal conditions and Table 3 by non-musculoskeletal conditions) details these findings.

**Table 2.** Summary of findings by **musculoskeletal condition** represent our recommendations and research direction across the 1) level of evidence from a scoping review, 2) psychometric properties from two psychometric property reviews, 3) expert Delphi survey data, 4) taskforce recommendation as appropriate for clinical use, and 5) future research priority. The Delphi table includes only the relevant ages Table 2.

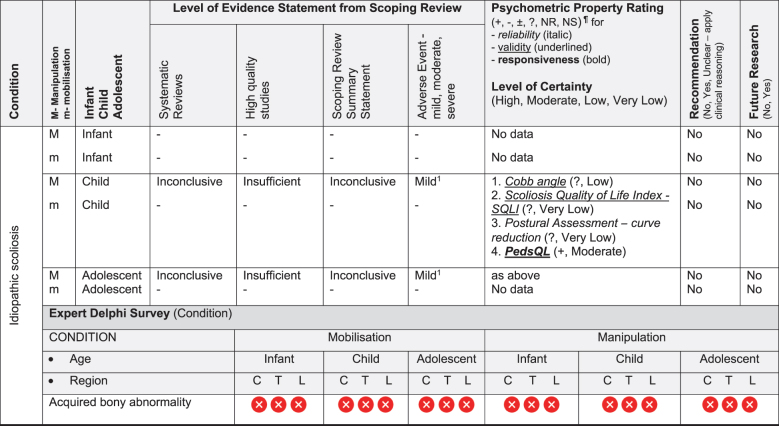
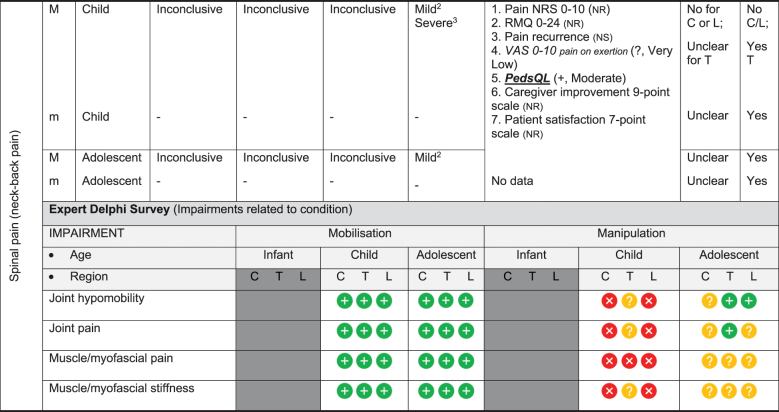
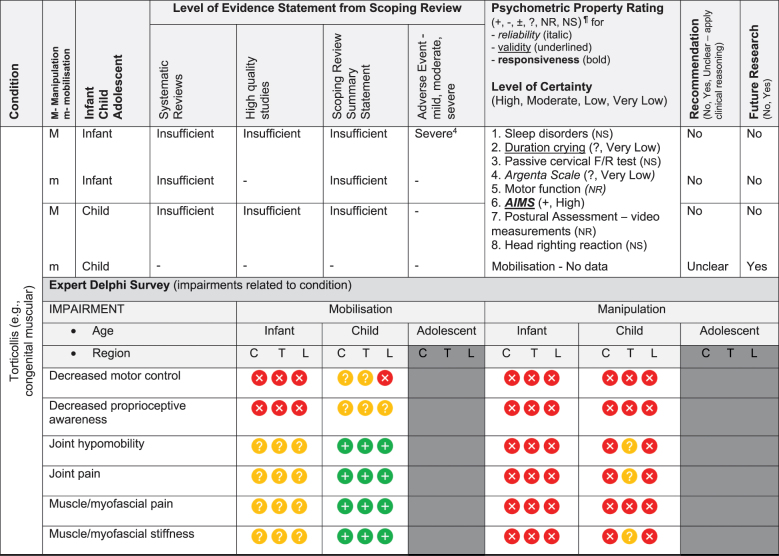
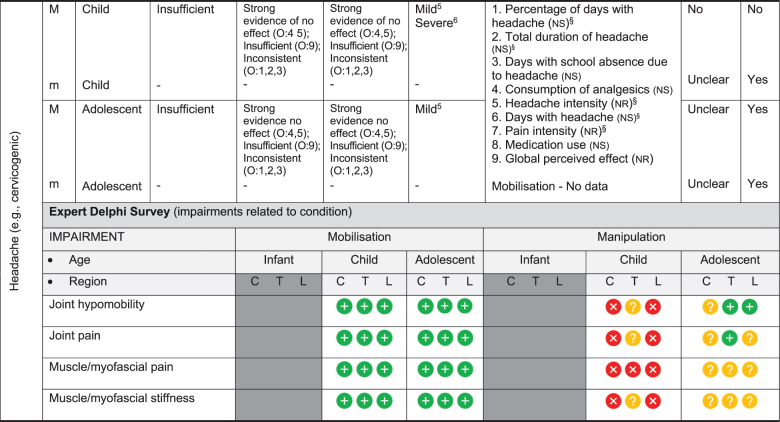
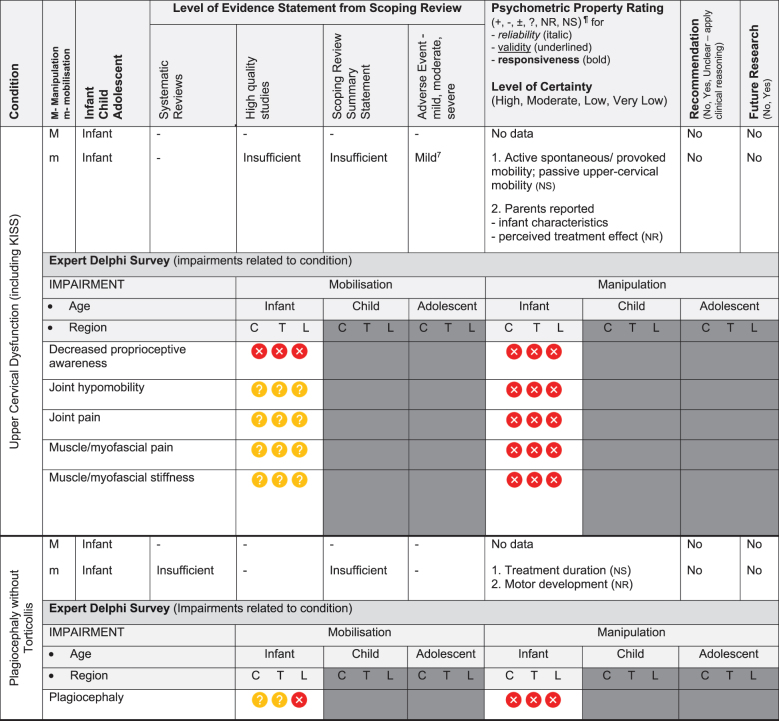

KEY: Infant (birth to <2 years); Child (2 to 12 years); Adolescent (13 to < 18 years); 7 or 9-pt = 7 or 9-point scale; AE = Adverse Events; AIMS = Alberta Infant Motor Scale; passive F/R test = passive flexion/ rotation test; KISS = kinematic imbalance due to suboccipital strain; NRS = numeric rating scale; VAS = visual analogue scale; PedsQL = Pediatric Quality of Life Inventory; RMQ = Roland and Morris Questionnaire; C = Cervical; T = Thoracic; L = Lumbar. **Levels of evidence rating (scoping review)**: very strong, strong, moderate, limited evidence; **Summary Statement**: ‘inconclusive’ = If ≥ 66.6% of relevant investigations were not reached and results of the decision tree were mixed, ‘insufficient’ = If there were insufficient studies/reviews exploring the intervention for the identified condition.

**Delphi:** 
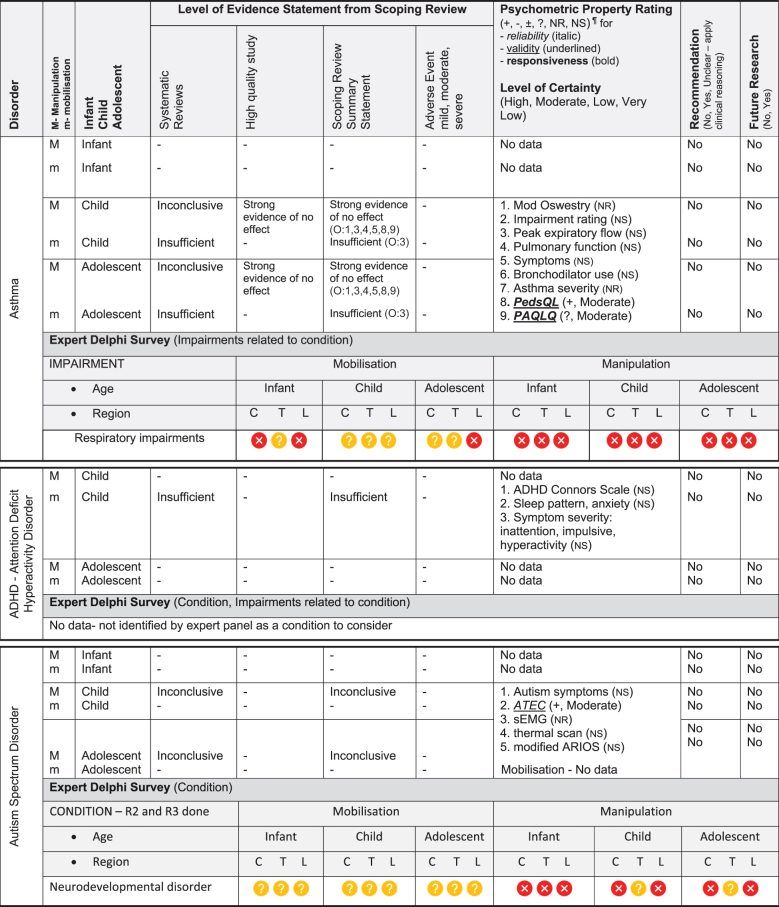
  = Consensus that the treatment (mobilisation or manipulation) is NOT appropriate (impairments) or NOT recommended (condition) for infant/child/adolescent; 
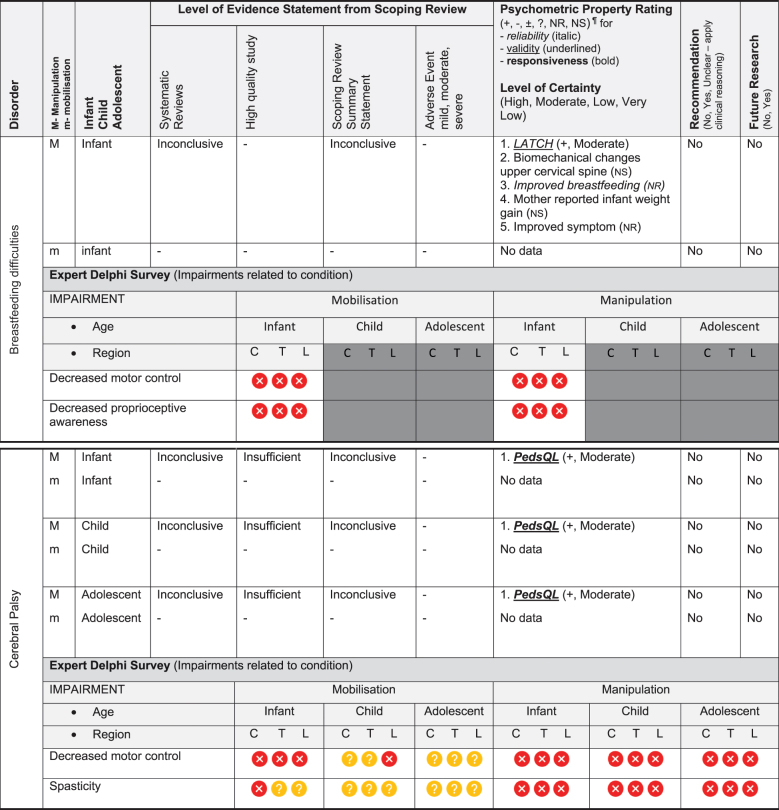
 = No consensus; 
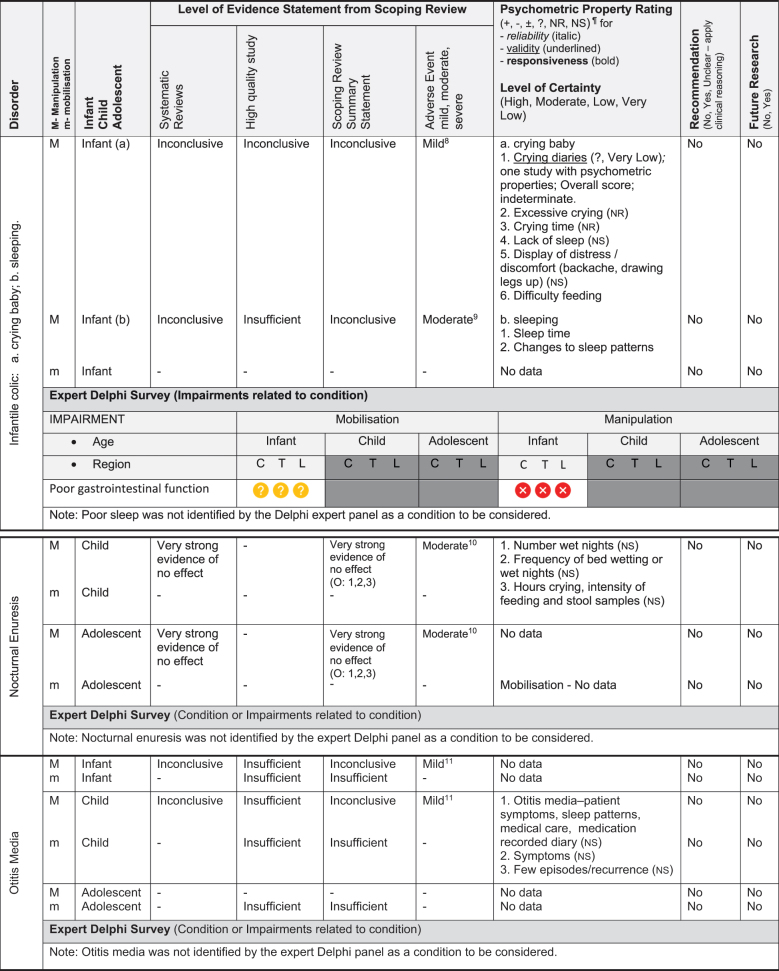
 = Consensus that the treatment (mobilisation or manipulation) may be appropriate (impairment) or recommended (condition) for infants/children/adolescents.

**¶Psychometric Property Criteria Rating**: (+) = sufficient, (−) = insufficient, (±) = inconsistent, (?) = indeterminate, (NR) = searched for but not reported [14,23], (NS) = no search performed. **Level of Certainty (for clinical outcome measure) using GRADE approach**: H = high certainty evidence,  M = moderate certainty evidence, L = low certainty evidence, VL = very low certainty evidence.

§ Guidelines of the International Headache Society: Clinical outcome assessments tools should include improvements in severity/duration/frequency of headache and Quality of life [27,28]. **Adverse Event** (AE) documented in studies from the scoping review as Mild, Moderate, and Severe follow [17]: ^*1*^***Mild AE*** = Two benign reactions (no further detail documented, chiropractic) [29]; ^*2*^***Mild AE*** = randomised controlled trial, unusual treatment soreness and different type of pain [30]; ***^3^Severe AE*** = Unsteady gait, poor coordination, drowsiness, hospitalisation with delayed diagnosis of congenital occipitalisation in a 12-year-old girl with history of congenital torticollis [31], progressive deficits in legs, clonus at rest, urinary urgency, paraplegia in 12 year old girl with history of osteogenesis imperfecta [32]; ^*4*^***Severe AE*** = subarachnoid hemorrhage and death; this case report was of a 3 month old girl [33]; quadra or paraplegia; this was a case report of a 4 month old boy astrocytoma [34]; ^*5*^***Mild AE*** = Hot skin and dizziness, transitory increase in headache intensity & frequency; quick recovery once treatment stopped; this randomised controlled trial had 52 children age 7 to 15 years old; the trial was stopped early due to frequency of complaints [35]; ***^6^Severe AE*** = Severe occipital and bifrontal headache, vomiting, left facial weakness, diplopia, ataxia; this case report was of a 7 year old boy gymnast [36]; ^*7*^***Mild AE*** = Frequent (14%) mild AE (i.e. back soreness, irritability, poor feeding, mild distress, increased crying or head tilt, temporary vegetative responses); this prospective cohort study included 307 infants (<27 weeks) with upper cervical dysfunction [37]

### Literature review stage

The scoping review [17] included 87 studies (35 systematic reviews, 16 randomised trials, 11 other studies such as cohort, 2 guidelines, 14 opinion papers, and 9 policy papers) describing the use of spinal manipulation and mobilisation for 14 paediatric conditions by four health professional groups – physicians, physiotherapists, chiropractors, osteopaths. Most of these conditions (8/14) were non-musculoskeletal and had no evidence, inconclusive evidence, or insufficient evidence to support the use of spinal manipulation or mobilisation to treat these conditions. Tables 2 and 3 report the levels of evidence findings from systematic reviews, high quality studies, and adverse event papers by condition. There was strong to very strong unfavourable evidence for the use of spinal manipulation for the management of asthma, headache, and nocturnal enuresis. Adverse events were commonly described to be mild, but moderate to severe adverse events were reported in some systematic reviews and low-quality studies.

**Table 3.** Summary of findings by non-musculoskeletal condition represent our recommendations and research direction across 1) level of evidence from a scoping review, 2) psychometric properties from two reviews, 3) expert Delphi survey data, 4) taskforce recommendation as appropriate for clinical use, and 5) future research priority.

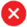
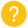
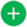

KEY: Infant (birth to <2 years 18); Child (2 to 12 years); Adolescent (13 to <18 years); 7 or 9-pt = 7 or 9-point scale; ADHD = Attention Deficit Hyperactivity Disorder (age 6 to 18); ARIOS = Autism research institute outcomes survey; ASD = Autism Spectrum Disorder; ATEC = Autism treatment evaluation checklist; CNS = central nervous system; sEMG = surface electromyography for autism spectrum disorder; LATCH = Latch, Audible swallowing, Type of nipple, Comfort, Hold; PAQLQ = Pediatric Asthma Quality of Life Questionnaire; C = Cervical; T = Thoracic; L = Lumbar; **Levels of evidence rating (scoping review):** very strong, strong, moderate, limited evidence. **Summary Statement:** ‘inconclusive’ = If ≥ 66.6% of relevant investigations were not reached and the results of the decision tree were mixed, ‘insufficient’ = if there were insufficient studies/reviews exploring the intervention for the identified condition/outcome.**Delphi:** 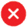  = Consensus that the treatment (mobilisation or manipulation) is NOT appropriate (impairments) or NOT recommended (condition) for infant/child/adolescent; 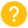  = No consensus;  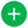 = Consensus that the treatment (mobilisation or manipulation) may be appropriate (impairment) or recommended (condition) for infants/children/adolescents. ¶**Psychometric Property criteria rating:** (+) = sufficient, (−) = insufficient, (±) = inconsistent, (?) = indeterminate, (NR) = searched for but no report [14;23,] (NS) = no search performed; **Level of Certainty (clinical outcome measure) using GRADE approach:** H = high certainty evidence, M = moderate certainty evidence, L = low certainty evidence, VL = very low certainty evidence. **Adverse Event (AE)** based on scoping review [17]; ^8^***Mild AE*** = Increased crying in a cohort study of 158 infants categorised into three subgroups, (A) infant colic, (B) irritable infant syndrome of musculoskeletal origin (IISMO) and (C) inefficient feeding crying infants with disordered sleep (IFCIDS) [38]; ^9^***Moderate AE*** = 53% vegetative reaction - flushing, reflex apnoea, hyperextension, sweating, bradycardia, tachycardia; this cohort study was of 199 infants average age 5-months receiving gentle manipulation impulse (<5N) [39]; rib fracture; this case report of posterior rib fractures was in a young infant [40]; ^10^***Moderate AE*** = This randomised controlled trial reported development of severe headache, stiff neck, acute lumbar spine pain [41]; ^11^***Mild AE*** = Increased irritability and mid-back soreness; this feasibility randomised controlled trial of 20 participants was conducted on infant (6-months) to children aged 6-years[42].

The psychometric properties of patient-reported, observer-reported, clinician-reported, and performance-based clinical outcome measures identified 95 studies assessing 10 outcome measures [18,19]. Tables 2 and 3 indicate the clinical outcome assessments with reliable (italicised), valid (underlined), and responsive (bolded) psychometric property ratings. The clinical outcome assessment with sufficient measurement properties were PedsQL (Pediatric Quality of Life Inventory) for measuring quality of life in paediatric populations with asthma, cerebral palsy, idiopathic scoliosis, neck-back pain (moderate certainty evidence); Alberta Infant Motor Scale (AIMS) for assessing motor development in infants with congenital torticollis (high certainty evidence); Autism Treatment Evaluation Checklist (ATEC) for Autism related symptoms in children with autism spectrum disorder (moderate certainty evidence); and LATCH (Latch, Audible swallowing, Type of nipple, Comfort, Hold) for breastfeeding in healthy infants (moderate certainty evidence). The remainder of the paediatric conditions and impairments investigated by the taskforce lacked clinical outcome assessments for paediatric populations with sufficient psychometric properties.

### Delphi stage

A total of 26 international physiotherapists from seven countries and five continents with paediatric or orthopedic manipulative therapy expertise participated and 11 completed all 3-Rounds. There were several conditions in the scoping review that were not identified by the Delphi expert panel as appropriate for spinal manipulation and mobilisation including nocturnal enuresis, otitis media, infantile colic (other than poor gastrointestinal function), and Attention Deficit Hyperactivity Disorder (ADHD). Due to variations in terminology between the scoping review and the Delphi, there were other conditions that were not specifically identified in the Delphi but had associated impairments to treat identified by the Delphi panel after Round-1 including cerebral palsy, breastfeeding difficulties, asthma, headache, torticollis, spinal pain and upper cervical dysfunction in infants including Kinetic Imbalance due to Suboccipital Stress (KISS). The impairments in the Delphi study that were associated with each condition were considered by the taskforce. Autism was included under the umbrella term neurodevelopmental disorder in the Delphi study. Conditions such as headache and neck-back pain were excluded for infants; the taskforce determined that such diagnoses for infants were not plausible.

**Table 4.** Evidence-based practice position statement on spinal manipulation and mobilisations for paediatric populations predicated on clinicians using biopsychosocial clinical reasoning to determine when the intervention is appropriate.

Directive	Evidence-based practice position statement
**NOT** **RECOMMENDED** (do not perform)	Spinal manipulation and mobilisation should not be performed on infants. Cervical and lumbar spine manipulation should not be performed on children. Spinal manipulation and mobilisation are not appropriate and should not be performed to treat non-musculoskeletal conditions among infants, children and adolescents including asthma, attention deficit hyperactivity disorder, autism spectrum disorder, breastfeeding difficulties, cerebral palsy, infantile colic, nocturnal enuresis, and otitis media.
May be **APPROPRIATE** with sound clinical reasoning	Spinal mobilisation may be appropriate to treat children with musculoskeletal conditions including spinal mobility impairments associated with neck-back pain, and neck pain with headache. Thoracic spine manipulation may be appropriate to treat children with musculoskeletal conditions including spinal mobility impairments associated with neck-back pain. Spinal manipulation and mobilisation may be appropriate to treat adolescents with musculoskeletal conditions including spinal mobility impairments associated with neck-back pain and neck pain with headache.
**RECOMMEND**	No high certainty evidence is available to recommend spinal mobilisation or manipulation for paediatric populations.

### Refinement stage: position statements

Through a series of online consensus workshops, the taskforce engaged in extensive discussion, debate, and synthesis to develop consensus on the final evidence-based practice position statements noted in Table 4. The overarching summative analysis of the data created by the taskforce resulted in three evidence-based practice position statements where spinal manipulation and mobilisation were not recommended, three position statements where spinal manipulation or mobilisation may be appropriate when supported by sound clinical reasoning and one position statement identifying no evidence of high certainty was available to recommend manipulation or mobilisation with confidence (see Table 4). The underpinning details are based on the summary of finding tables (see Tables 2 and 3) and reported for each evidence-based practice position statement as follows.

### Not recommended

- ***Spinal manipulation and mobilisation should not be performed on infants.***

There was insufficient evidence on mobilisation for managing torticollis, upper cervical dysfunction-KISS, plagiocephaly, and otitis media. Only one clinical outcome assessment – AIMS for torticollis – was identified as reliable, valid, and responsive. The Delphi survey [24] of expert international physiotherapists (i.e., Delphi panel) demonstrated consensus that manipulation is not recommended for infants across all conditions, impairments, and spinal levels. Spinal mobilisation was determined to be not appropriate to treat infants for any condition except neurodevelopmental disorders where there was no consensus. Further, torticollis is a condition affecting infants and due to insufficient evidence and safety concerns, the taskforce did not recommend use of manipulation or mobilisation for infants with torticollis. The taskforce judgment was to not recommend (do not perform) spinal manipulation or mobilisation in infants for three reasons: (1) the adverse events in infants ranged from mild (i.e., temporary vegetative response) to severe (i.e., death) across conditions; (2) spinal manipulation and mobilisation had insufficient evidence; and (3) spinal manipulation and mobilisation were not recommended for infants across all conditions by the Delphi expert panel. We acknowledge reporting of adverse events in clinical trials was poor and the risk rates could not be determined. These interventions may appear to be acceptable to carers and some healthcare professionals based on beliefs and their personal experience; however, serious safety concerns remain. The adverse events are more likely to occur if there is exposure to manipulation or mobilisation, and these adverse events are unacceptable (i.e., death) when other alternative and effective treatment choices are available.

We acknowledge that we had strict age cut points in our definitions of infant and child during the review and Delphi stage. However, through discussion and integration during the refinement stage of biopsychosocial perspectives, specifically cognitive development theory [43,44] of a young child (<7 years, preoperational stage) and infant (<2 years; sensorimotor stage), we identified cognitive abilities, language usage, and physical growth to be important biopsychosocial determinates in the clinical reasoning process for the judgment of the application and safe use of spinal manipulation and mobilisation. For this reason, it was the taskforce’s opinion that the clinician may wish to extend the age of this directive to a young child (<7 years). No further research regarding spinal manipulation or mobilisation for infants was recommended.

- ***Cervical and lumbar spine manipulation should not be performed on children.***

For children with neck-back pain conditions, the evidence regarding spinal manipulation was inconclusive and the evidence regarding spinal manipulation for managing torticollis in children was insufficient based on systematic reviews and high-quality studies. Only one clinical outcome assessment – the PedsQL – was identified as reliable, valid, and responsive in neck-back pain and AIMS in torticollis. There were adverse effects reported in the reviewed literature that ranged from mild symptoms such as unusual treatment soreness, to severe adverse events such as unsteady gait, poor coordination, urinary incontinence, and paraplegia. Equally for neck pain with headache, there was insufficient evidence from systematic reviews but strong evidence of no effect from high quality studies. There was also evidence of mild and severe adverse events (loss of consciousness, left facial weakness, diplopia, ataxia) for cervical manipulation performed on children to treat chronic headache from the descriptive synthesis of high-quality studies. Therefore, the taskforce followed the Delphi panel consensus that spinal manipulation was not recommended for clinical use in children at cervical and lumbar levels and in children with neck-back pain, torticollis, or headache.

- ***Spinal manipulation and mobilisation are not appropriate and should not be performed to treat non-musculoskeletal conditions among infants, children and adolescents including asthma, attention deficit hyperactivity disorder, autism spectrum disorder, breastfeeding difficulties, cerebral palsy, infantile colic, nocturnal enuresis, and otitis media.***

There was strong to very strong evidence for no significant effect of spinal manipulation for managing asthma and nocturnal enuresis in children and adolescents [17]. The outcome for asthma was based on only one reliable, valid, and responsive clinical outcomes assessment – the PedsQL. There was a lack of validated clinical outcome assessment for enuresis. Additionally, there was inconclusive evidence (autism, breast feeding difficulties, cerebral palsy, infantile colic – manipulation, otitis media – manipulation) and insufficient evidence (asthma – mobilisation; ADHD, autism, otitis media – mobilisation) in infants, children, or adolescents. The PedsQL was identified as a reliable, valid, and responsive clinical outcome measure for use with paediatric populations with in cerebral palsy and asthma as well. The LATCH in breastfeeding difficulties and ATEC in autism were reliable and valid but lacked evidence on responsiveness. The crying diaries in infantile colic had indeterminate validity of very low certainty evidence. For the conditions that had valid and responsive clinical assessment psychometric properties, the results of the scoping review can be accepted with moderate certainty. For non-musculoskeletal conditions, the Delphi panel either reached consensus that spinal mobilisation or manipulation were not appropriate (breastfeeding difficulties), did not identify the condition as one to be considered (otitis media, nocturnal enuresis, ADHD), or lacked consensus on appropriateness (asthma/respiratory impairments, neurodevelopmental disorders, cerebral palsy, infantile colic). In the non-musculoskeletal conditions where the Delphi panel lacked consensus, the taskforce made the determination of not recommended due to lack of neurophysiological and biomechanical plausibility. This lack of plausibility combined with strong evidence of no effect (asthma, nocturnal enuresis), no evidence (ADHD), inconclusive (cerebral palsy, autism, breastfeeding difficulties, infantile colic), or insufficient (ADHD, otitis media) evidence based on findings from the scoping review identified that spinal manipulation or mobilisation were not clinically indicated. There were conditions where the Delphi panel lacked consensus on impairments related to conditions (respiratory impairments, neurodevelopmental disorders, decreased motor control, spasticity, and poor gastrointestinal function) and the taskforce made the determination of not recommended due to safety concerns such as reflex apnoea, bradycardia, tachycardia, vegetative reactions, and rib fracture combined with insufficient or lack of evidence from the scoping review.

### May be appropriate

- ***Spinal manipulation and mobilisation may be appropriate to treat adolescents with musculoskeletal conditions including spinal mobility impairments associated with neck-back pain and neck pain with headache.***

Spinal manipulation to treat adolescents with neck-back pain had inconclusive evidence and neck pain with headache had insufficient evidence from review of systematic reviews, while there were no data in other words, no evidence on spinal mobilisations. The descriptive synthesis of high-quality studies on manipulation for neck-back pain was inconclusive and for headache indicated strong evidence of no effect for specific outcomes (i.e., consumption of analgesics, headache intensity) however inconsistent evidence exists for other outcomes (i.e., percentage of days with headache, total duration of headache, and days with school absence due to headache). Additionally insufficient evidence was identified for the outcome global perceived effect and no evidence on quality of life, a clinical outcome assessment recommended by International Headache Society [27]. For neck-back pain, of the seven clinical outcome assessments used in the scoping review for neck-back pain, only PedsQL was identified to be reliable, valid, and responsive. The taskforce felt that the indirectness of the data influenced our findings as follows:

1. For both neck-back pain and neck pain with headache, findings emerged from data that spanned two age periods (child and adolescents). Direct data specific to each age group is needed in future trials;
2. For neck pain with headache, we noted a variance in the manipulation technique and dose from a single session to 8-sessions over 16 weeks;
3. For neck pain with headache, there was unclear classification of headache type, subtype, and form within and between studies. Participants with mixed headache groups were frequently reported (i.e., ‘recurrent headache’) without further diagnostic classification consistent with the International Classification of Headache Disorders [28].

The evidence for spinal manipulation to treat neck-back pain was inconclusive with reports of mild adverse events for adolescents. The Delphi panel had no consensus for cervical manipulation in adolescents for mobility and pain impairments, but consensus was reached to support use of thoracic and lumbar manipulation for the joint hypomobility. Additionally, the Delphi panel did support the use of mobilisation to treat mobility and pain impairments in adolescents for cervical, thoracic, and lumbar regions. After much consideration, the taskforce consensus was spinal manipulation and mobilisation may be appropriate with the use with sound clinical reasoning to treat spinal mobility impairments for neck-back pain and neck pain with headache in adolescents. There is a need for monitoring through (1) ongoing systematic reviews of benefits and harms, and perhaps most importantly (2) mandatory reporting to regulatory bodies to establish a better estimate of harms. Well conducted phase-2 or phase-3 clinical trials that use valid and responsive clinical outcome assessments would add clarity for future evidence-based recommendations on the use of spinal manipulation to treat spinal pain.

- ***Spinal mobilisation may be appropriate to treat children with musculoskeletal conditions including spinal mobility impairments associated with neck-back pain and neck pain with headache.***

Spinal mobilisation to treat children with neck-back pain and neck pain with headache had no evidence of effectiveness or adverse events from the scoping review. The Delphi panel had reached consensus favouring the use of spinal mobilisation to treat spinal mobility impairments associated with spinal pain. The taskforce’s recommendation was spinal mobilisation may be appropriate with the use of sound clinical reasoning in children to treat spinal impairments. However, the taskforce extends a similar caution and has concerns regarding the unclear risk of adverse events when mobilisations are applied to a young child (<7 years) as previously noted. Further research is recommended for these musculoskeletal conditions and spinal impairments.

- ***Thoracic spine manipulation may be appropriate to treat children with musculoskeletal conditions including spinal mobility impairments associated with neck-back pain.***

While the evidence from the scoping review was inconclusive for spinal manipulation in children with mild and severe adverse events noted, the Delphi panel did not reach consensus for thoracic manipulation. Of the seven clinical outcome assessments used in the scoping review for neck-back pain, only PedsQL was identified to be reliable, valid, and responsive. The taskforce recommendation was that thoracic manipulation may be appropriate with the use of sound clinical reasoning to treat children with spinal mobility impairments associated with neck-back pain.

### Recommended

- ***No evidence of high certainty is available to recommend spinal manipulation or mobilisation for paediatric populations.***

### Evidence-based gap analysis

**Figure 2.**
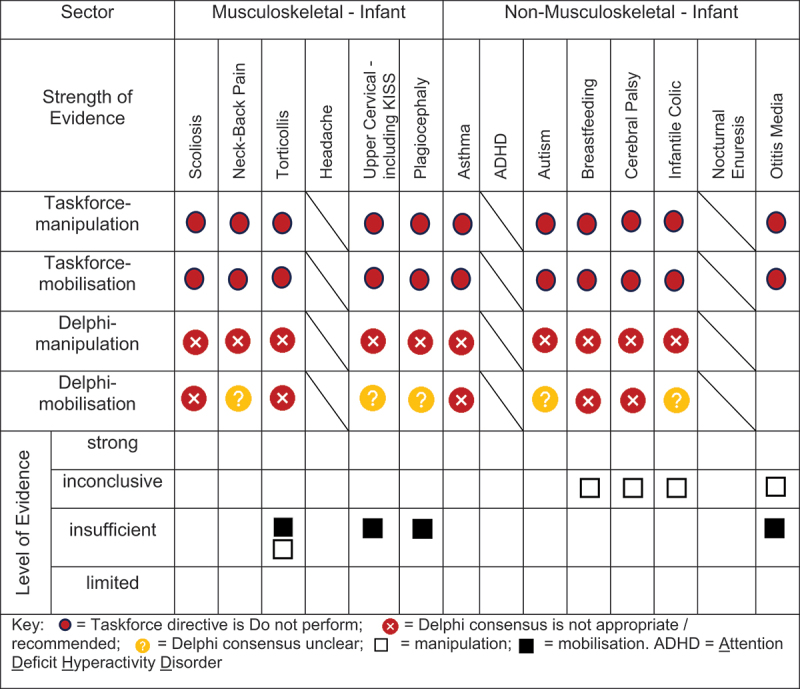
Evidence gap map for INFANTS by musculoskeletal and non-musculoskeletal condition.

**Figure 3.**
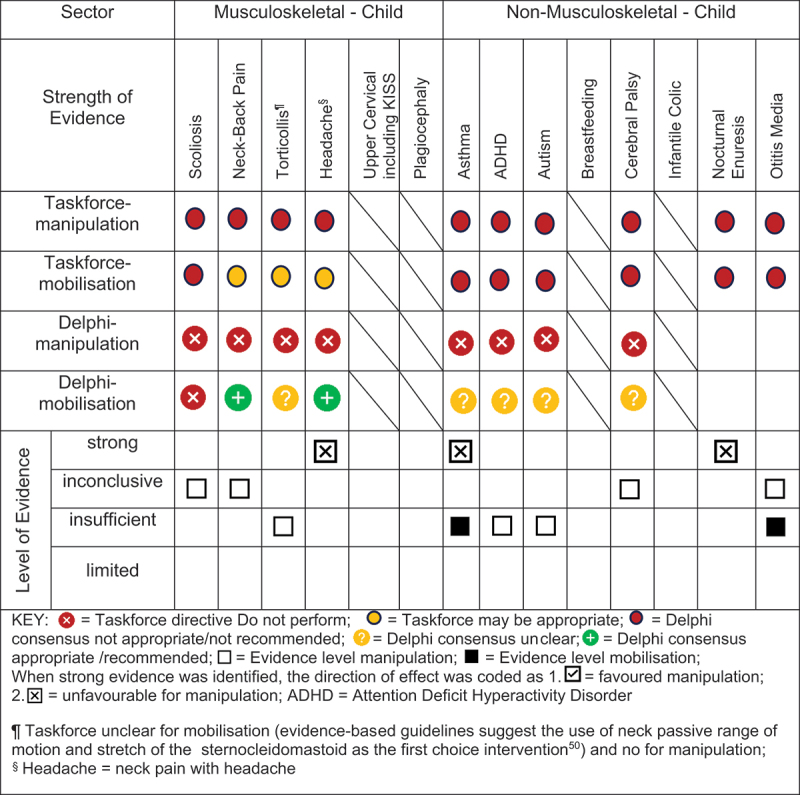
Evidence gap map for CHILDREN by musculoskeletal and non-musculoskeletal condition.

**Figure 4.**
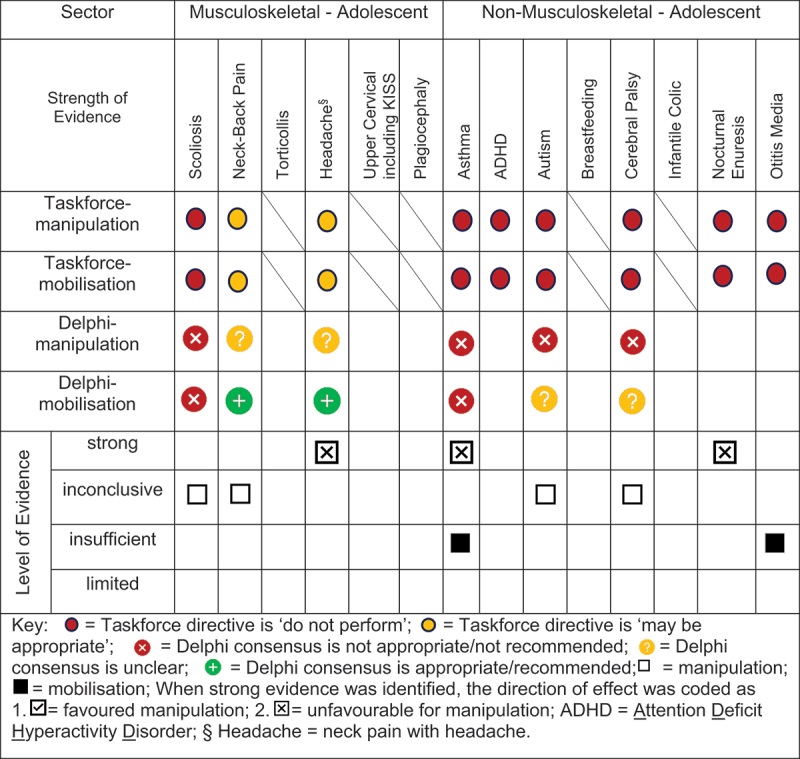
Evidence gap map for ADOLESCENTS by musculoskeletal and non-musculoskeletal condition.

We developed evidence-based gap maps for infants (Figure 2), children (Figure 3), and adolescents (Figure 4). Three research themes detailed in the discussion section were identified:

- priority paediatric conditions,
- psychometric properties,
- adverse events.

Evidence-based gaps exist based on our literature review for non-musculoskeletal conditions in infants, children, and adolescents including no evidence (ADHD); inconclusive (cerebral palsy, autism, breastfeeding difficulties, infantile colic); insufficient (ADHD, otitis media); and strong evidence of no effect (asthma, nocturnal enuresis). Both the expert Delphi panel and the taskforce of clinician-scientists identified that spinal manipulation or mobilisation were not clinically indicated in non-musculoskeletal paediatric conditions. In short, the foundational biological and neurophysiological rationale underpinning their clinical use should be scientifically established prior to use in clinical trials; this evidence must establish clear plausibility before using valuable research resources to conduct well designed large cohort or randomised controlled trials. Equally so, evidence-based gaps exist for musculoskeletal conditions and impairments in infants for the use of manipulation and mobilisation as follows: no evidence (idiopathic scoliosis, neck-back pain) and insufficient evidence (torticollis, upper cervical dysfunction including KISS, plagiocephaly) (Figure 2). No further research or clinical use is recommended due to safety concerns identified in cohort trials and case reports. Additionally, clinical practice guidelines recommend viable and safe alternative approaches detailed in the discussion section for all but two of these conditions. Finally, evidence-based gaps exist in children for musculoskeletal conditions, specifically scoliosis, torticollis, plagiocephaly, neck-back pain, and neck pain with headache (Figure 3). The latter had strong evidence of no effect (headache). Practice guidelines exist for scoliosis [45], torticollis [46], and plagiocephaly [47] identifying viable safe alternative treatment approaches and these guidelines do not recommend manipulation or mobilisation. Future research is needed on spinal mobilisation in children and adolescents and on spinal manipulation in adolescents for neck-back pain and neck pain with headache. Further research and clinical use were not recommended for cervical or lumbar manipulation in children due to safety concerns.

Evidence gaps for clinical outcome assessments were identified by paediatric condition in Tables 2 and 3; 57% (8/14) of the conditions in the literature review stage had a single clinical outcome assessment with a sufficient psychometric property criteria rating of moderate certainty for reliability and validity (idiopathic scoliosis, neck-back pain, torticollis, asthma, autism spectrum disorder, breastfeeding dysfunction and cerebral palsy) while 36% (5/14) of the conditions used two clinical outcome assessments (AIMS, PedsQL) with sufficient evidence of responsiveness. This evidence-based gap will require further research to establish valid/responsive clinical outcome assessments for conditions where spinal manipulation and mobilisation may be appropriate.

Perhaps the *highest* research priority was the inclusion of standard documentation and reporting of adverse events in paediatric clinical trials on spinal manipulation and mobilisation; Tables 2 and 3 depict limited reporting; 50% of conditions had evidence of some adverse events notated. Severe adverse events did not appear in randomised trials but in other lower quality studies and systematic reviews. Severe adverse events were reported following manipulation for neck-back pain (child), torticollis (infant), and headache (child); moderate adverse events were reported following manipulation for infantile colic and nocturnal enuresis (child/adolescent) and mild adverse events following manipulation for scoliosis (child/adolescent), neck-back pain (child/adolescent), headache (child/adolescent), colic (infant/child), and otitis media (infant/child). No evidence of severe or moderate adverse events were reported following spinal mobilisation; however, mild adverse events were reported following mobilisation for upper cervical dysfunction including KISS (infant).

## Discussion

Seven evidence-based position statements emerged identifying parameters for when spinal manipulation and mobilisation should not be performed or may be appropriate. The most compelling evidence and resulting position statements state that spinal manipulation and mobilisation should not be performed on infants or to treat non-musculoskeletal paediatric conditions among infants, children, and adolescents. Additional statements were identified that spinal manipulation and mobilisation may be appropriate to treat adolescents with musculoskeletal conditions including spinal mobility impairments associated with neck-back pain and neck pain with headache; or spinal mobilisation or thoracic spine manipulation to treat children with spinal mobility impairments.

Available evidence was often insufficient or inconclusive, and this, combined with inadequate psychometric properties of the clinical outcome assessments used in the reviewed clinical trials, necessitated use of expert opinion from a Delphi study combined with taskforce consensus workshops to develop the position statements. To further inform recommendations for priority research, detailed discussion held by the taskforce on the evidence gaps identified during the synthesis and summative analysis follows.

- *Musculoskeletal paediatric conditions – Neck-back pain or neck pain with headache*

A research focus on spinal mobilisation techniques for children and manipulation-mobilisation techniques for adolescents with musculoskeletal conditions including spinal mobility impairments associated with neck-back pain and neck pain with headache is needed to test the recommendations of the taskforce and to develop future clinical guidelines. A full systematic review and meta-analysis on this topic for spinal pain and headache in paediatric populations is underway [48]. Clinical outcome assessments must be developed with sound psychometric properties and a priority outcome set identified to proceed with this line of research. A phase-2 dose trial is needed prior to embarking on a larger definitive phase-3 well-designed (factorial design) dose trial. Research must include development and testing of knowledge translation tools to guide parents and carers toward an evidence-based informed decision-making process. However, further research is not recommended on spinal manipulation for infants and children (cervical/lumbar) or mobilisation on infants due to the unknown but related risk of adverse events in a developing spine. Prevention (manipulation on healthy children) accounted for the largest portion of manipulations in children younger than 4-years [49] yet guidelines do not recommend use of spinal manipulation in infants and young children nor is there evidence to support use of this intervention to prevent future spinal conditions or impairments. Decisional needs assessment and decision aids may help the parent/carer/child in determining their best clinical treatment pathway during shared decision-making processes.

- *Musculoskeletal paediatric conditions – Scoliosis.*

The evidence-based findings on spinal manipulation and mobilisation for scoliosis in children and adolescents were inconclusive. In addition, the Delphi panel did not recommend spinal manipulation or mobilisation to treat bony abnormalities. Guidelines and systematic reviews do not support the use of spinal manipulation or mobilisation alone for this condition [45]. Research on this topic was not recommended.

- *Musculoskeletal paediatric conditions – Congenital torticollis, upper cervical dysfunction-KISS syndrome, and plagiocephaly.*

For congenital torticollis, there was insufficient evidence identified but the potential for adverse events was moderate to severe for spinal manipulation and not reported for spinal mobilisation. The related relative risk could not be established [17]. The Delphi expert panel identified that while spinal manipulation was not appropriate for infants and children, mobilisation may be appropriate for related impairments in children. The most recent clinical practice guidelines [46,50] recommend first-line treatment for congenital torticollis in infants to be neck passive range of motion with the focus of stretching the sternocleidomastoid muscle, neck and trunk active range of motion, development of symmetrical movement, environmental adaptations, and parent/carer education. Further research for either mobilisation or manipulation was not recommended due to the sufficient evidence supporting current clinical practice with infants, but mobilisation efficacy remains unclear in children and could be explored.

Both upper cervical dysfunction-KISS (Kinematic Imbalances due to Suboccipital Stress) syndrome and plagiocephaly in infants’ treatment with spinal manipulation or mobilisation showed insufficient evidence, mild adverse events following mobilisation, and unknown risk of serious adverse events. The expert Delphi panel clearly identified that spinal manipulation was not appropriate for these two conditions and reached no consensus for spinal mobilisation. The taskforce held an extensive discussion, debate, considered the acceptability to carers and healthcare providers about these conditions, and reached 100% consensus that no further primary research is recommended. Our rationale follows: upper cervical dysfunction-KISS syndrome is theorised to be an upper cervical spine malalignment or mobility ‘fixation’ in infants. It is described to include a positional preference of the head to one side such as a fixed posture toward lateral flexion and contralateral rotation with both passive and active range of motion deficits. It is further theorised that upper cervical dysfunction – KISS may be accompanied by the infant being unsettled, restlessness, having body asymmetry of the trunk-extremities (C-shaped), congenital torticollis, and deformational plagiocephaly [37]. Plagiocephaly, as it relates to otherwise normal infants, was identified to be present in 54% of the studied infants with upper cervical dysfunction [37]. Saedt and colleagues identified positional preference to be the most common reason (79%) for parents to seek care from a paediatric manual physiotherapist; this was followed by restlessness (61%) and abnormal head position (55%) [37]. Positional plagiocephaly is common in healthy infants and has an incidence rate of 48% in typically healthy infants (<12-months) to 50% in infants < 3-months old [51,52]. While upper cervical dysfunction – KISS-syndrome is purported to be caused by birth trauma [53] creating an upper cervical fixation, other factors such as testosterone level in male fetuses may accentuate muscular action and the occurrence of congenital torticollis [47]. Additionally, differential diagnosis is needed to rule out more serious and confounding diagnoses causing congenital torticollis such as tumor, extra muscular masses, fractured clavicle, neurological damage (e.g., cerebral palsy, brachial plexus injury), osteogenic asymmetry, and craniosynostosis as well as to rule in associated congenital muscular torticollis and plagiocephaly [54]. Early identification of infants at risk of congenital muscular torticollis and head asymmetries is essential; the rate of correction for cranial asymmetry decreases as the infants grows older (>3-months) as they gain head control and can reduce time with pressure on the occiput [55]. It is therefore essential to initiate consultation early when the infant is not progressing as anticipated and prevent delaying other appropriate management strategies [47]. In other words, there appear to be valid, effective, and accessible alternative approaches with low risk of harm.

The taskforce further considered a benefit-harm assessment for these medical conditions. Does the balance between the desirable and undesirable effect favour the intervention? There appears to be both direct and indirect risks and adverse events associated with cervical manipulation and mobilisation for infants. One clinical non-randomised controlled observational cohort study noted ‘vegetative reactions’ that they classified as a mild adverse event [37]. The taskforce’s collective clinical judgment classified this response as a red flag [39]. Of note, one fatal case study [33] of an infant who received upper cervical manipulation described a similar response of reflex apnoea, sweating profusely followed by a massive and fatal brain edema. In one cohort study of 199 infants, the authors reported a minor side effect as ‘a vegetative state’ in 54% of patients. Symptoms included reflex apnoea (<10 sec) in 22%, profuse sweating in 8%, flushing in 49% and bradycardia (up to 10 sec) in 42% [37]. The spinal mobilisation used in this case was identified to be a light manual pressure of about 11 Newtons by paediatric manual physiotherapists in The Netherlands. Thrust manipulation of 30 to 70 Newtons was avoided. However, other authors have advocated use of a ‘manipulation with an impulse’ directed to the upper cervical spine of infants to treat upper cervical dysfunction including KISS-syndrome [53]. The indirect associated risk includes the delayed diagnosis and initiation of more evidence-based interventions. These interventions might include stretching of the sternocleidomastoid via passive range of motion stretching exercises, environmental modifications, and potentially helmet therapy for managing congenital muscular torticollis with plagiocephaly [47,50]. The taskforce, therefore, did not identify sufficient foundational evidence to recommend conducting a large clinical randomised controlled trial or the clinical application of spinal manipulation or mobilisation for upper cervical dysfunction – KISS syndrome, congenital torticollis, or plagiocephaly.

- *Non-musculoskeletal paediatric conditions*

For infants, children and adolescents, there was either no evidence, insufficient, inconclusive evidence, or strong evidence of no benefit (asthma in adolescents) and neither the Delphi panel nor the taskforce could support the use of spinal manipulation or mobilisation for non-musculoskeletal conditions. Although it has been theorised that upper cervical dysfunction – KISS syndrome in infants creates a cascade of maladaptive physiological responses resulting in non-musculoskeletal conditions such as, but not limited to, colic, attention deficit disorder, and otitis media [53]; this hypothesised causal chain remains hypothetical and has not been established. There may appear to be evidence gaps, but the taskforce identified that foundational and plausible rationale for spinal manipulation and mobilisation use in these conditions were not well developed, and research, specifically further randomised controlled trials, in these areas was not recommended unless plausible biological rationale emerges. Evidence-gaps exist however must be underpinned by plausible biological rationale and sequenced research design.

- *Psychometric properties*

There was a large evidence gap in valid and responsive clinical outcome assessments [18,19]. Trialist should avoid use of outcome measures validated in adults only (i.e. Roland and Morris Questionnaire). Guidelines from the International Headache Society suggest the optimal clinical outcome assessment tools should include frequency of headache (i.e. monthly headache days) [56], headache severity, headache duration, and quality of life [27,28]. The psychometric properties evidence-based gap will require future research to identify, select, and validate core outcome sets for paediatric populations with musculoskeletal spinal conditions.

- *Adverse event analysis*

We recommended and require systematic reporting of mild or severe adverse events in future research [57]. The relative risk of a severe adverse event could not be determined from reported data and incidence of mild transient symptoms ranges from 0.3% (95% CI 0.06 to 1.82) to 22.22% (95% CI 6.32 to 54.74) [4,17]. It was not possible to provide an overall conclusion about the safety of spinal manipulation or mobilisation; small, randomised trials will not pick up uncommon events [17]. Adverse events were reported [17] as *severe* in infant torticollis [33,34] child neck-back pain [31,32], child headache [35,36]; and reported as *moderate* for infant colic [39]; and child/adolescent nocturnal enuresis [5]. Parents and carers must understand there is a risk albeit unknown of severe and moderate adverse events before they proceed to selecting spinal manipulation or mobilisation for spinal paediatric conditions (see Table 2 and 3). Above all else, clinicians must assure the safety of the patient by screening for red flags and refraining from interventions that jeopardise the patient’s health and well-being. The taskforce strongly recommends that researchers adhere to guidelines for reporting adverse events (PRISMA harms [57]: identifying unintended effects of an intervention; measuring their frequency; and identifying factors associated with the unintended effects (risk factors). As harms are often infrequent or rare, they are most measurable through systematic reviews and meta-analysis. Establishing mandatory national reporting and monitoring of adverse events by all healthcare providers utilising spinal manipulation or mobilisation on paediatric clients could be a meaningful approach to establishing the risk rates of severe adverse events following the application of spinal manipulation or mobilisation in paediatric populations but may face feasibility and legal challenges.

### Limitations of the review process

This evidence-based position paper used an iterative process recommended for developing guidelines in the field of physiotherapy. While we integrate appraised evidence with clinical expertise, we did not address client’s preferences, family-centered care, client/family informed consent or shared decision-making processes. Ultimately, the context of the clinicians’ experience and clinical pattern recognition combined with the perspective and expectations of the client and their family must be considered in the clinical reasoning process for each client [17,58–60]. The lack of reporting on harms in primary studies limited our assessment of the benefit-harm analysis.

### Agreement and disagreement with other studies and reviews

Our position statements agree with other systematic reviews from Safer Care Victoria [6], a global summit on safety in chiropractic manipulation [5] and an osteopathic manipulative treatment update for paediatric conditions [61]. All review updates identify very little evidence of patient harm and perhaps minor adverse events occurring more commonly in very young children. Low to very low-level evidence of little or no effect of manipulation across multiple non-musculoskeletal paediatric conditions was noted when compared to usual care or sham. We agree that manipulation for non-musculoskeletal conditions such as asthma, otitis media, cerebral palsy, attention deficit hyperactivity disorder, and musculoskeletal conditions such as torticollis, scoliosis, and plagiocephaly should not be recommended. We, however, disagree with a recent chiropractic guideline statement update that posits that ‘the absence of research evidence does not equate to evidence of absence and subsequent denial of care’ [62]. Our evidence-based position statements direct physiotherapists toward safer practice. The overarching statement identifies that the most vulnerable to risk of adverse events associated with spinal manipulation were infants and children, and with spinal mobilisation were infants, and as such are not appropriate for use in these populations.

Spinal manipulation and mobilisation in adult populations have been found to be most effective if combined with education and exercise to meet the patient’s specific needs [63–66]. Other more recent standards for low back pain (i.e., Clinical Care Standard in Australia 2023 [67] make no reference to paediatric populations and have no reference to manipulation or mobilisation. Exploring this combination of care would be a valuable research pathway in adolescents and children with spinal hypomobility or pain.

## Conclusion

We established evidence-based practice position statements to support physiotherapists in their clinical reasoning on the use of spinal manipulation and mobilisation for varied conditions and impairments in paediatric populations. Spinal manipulation and mobilisation on infants as well as cervical/lumbar manipulation on children should not be performed but may be appropriate for adolescents. Nor should spinal manipulation or mobilisation be performed to treat non-musculoskeletal paediatric conditions. Future research for children and adolescents with musculoskeletal conditions (neck-back pain) should include focused systematic reviews, high-quality cohort studies or clinical trials, assessment of psychometric properties of clinical outcome assessments, consistent reporting of adverse events, and development of knowledge translation tools to support parent-child evidence-informed shared-decision-making.

## References

[1] Huijbregts PA. Manual therapy in children: role of the evidence-based clinician. J Manual Manipulative Ther. 2006;14(1):7–9. doi: 10.1179/106698106790820881

[2] Chiropractic Board of Australia. Interim policy on spinal manipulation for infants & young children. 2019 Codes and guidelines. 2019 March 14. Available from: https://www.chiropracticboard.gov.au/

[3] Chiropractic Board of Australia. Statement on paediatric care. Codes And Guidelines. 2017 Jun 22. Available from: https://www.chiropracticboard.gov.au

[4] Corso M, Cancelliere C, Mior S, et al. The safety of spinal manipulative therapy in children under 10 years: a rapid review. Chiropr Man Therap. 2020;28(1):12. 10.1186/s12998-020-0299-y PMC7041232 32093727

[5] Cote P, Hartvigsen J, Axen I, et al. The global summit on the efficacy and effectiveness of spinal manipulative therapy for the prevention and treatment of non-musculoskeletal disorders: a systematic review of the literature. Chiropr Man Therap. 2021;29(1):8. 10.1186/s12998-021-00362-9 PMC7890602 33596925

[6] Safer Care Victoria. Chiropractic spinal manipulation of children under 12- Independent review. Best Practice & Improvement. 2019. March 8. Available from: https://www.safercare.vic.gov.au/

[7] Green S, McDonald S, Murano M, et al. Systematic review of spinal manipulation in children: review prepared by cochrane Australia for safer care victoria. Melbourne, Victoria: Victorian Government; 2019.

[8] Australian Physiotherapy Association. No place for spinal manipulation in infant care. APA Media. 2020. https://australian.physio/media/no-place-spinal-manipulation-infant-care

[9] Straus SE, Glasziou P, Richardson WS, et al. Evidence-based medicine: how to practice and teach it. 5th ed. North York, Canada: Elsevier; 2019.

[10] Hoffmann T, Bennett S, Del Mar C. Evidence-based practice across the health professions. 4th ed. Chatswood, NSW, Australia: Elsevier Health Sciences; 2023.

[11] Rushton A, Beeton K, Landendoen J, et al. International federation of orthopaedic manipulative physical therapists educational standards in orthopaedic manipulative therapy. Part A: educational standards 2016: glossary of terms 2019 [cited 2019 Nov 17]

[12] National Cancer Institute. NCI guidelines for investigators: adverse event reporting requirements guideline. 2013.

[13] World Health Organization. WHO guidelines on basic training and safety in chiropractic. Geneva: WHO Press; 2005. https://iris.who.int/handle/10665/43352

[14] Mokkink LB, Prinsen C, Patrick DL, et al. COSMIN methodology for systematic reviews of patient-reported outcome measures (PROMs). User manual. (version 1.0). Feb ed. Amsterdam, The Netherlands: VU University Medical Center, 2018.

[15] Moher D, Schulz KF, Simera I, et al. Guidance for developers of health research reporting guidelines. PloS Med. 2010;7(2):e1000217. doi: 10.1371/journal.pmed.1000217 20169112 PMC2821895

[16] Alonso-Coello P, Schünemann HJ, Moberg J, et al. GRADE Evidence to Decision (EtD) frameworks: a systematic and transparent approach to making well informed healthcare choices. 1: introduction. Br Med J. 2016;353. doi: 10.1136/bmj.i2016 27353417

[17] Milne N, Longeri L, Patel A, et al. Spinal manipulation and mobilisation in the treatment of infants, children, and adolescents: a systematic scoping review. BMC Pediatr. 2022;22(1):721. doi: 10.1186/s12887-022-03781-6. published Online First: 271 36536328 PMC9762100

[18] Hayton T, Gross A, Basson A, et al. Psychometric measurement properties of patient-reported and observer-reported outcome measures for spinal mobilisations and manipulation on paediatric subjects with diverse medical conditions: a systematic review. J Manual Manipulative Ther. 2023;1–21. doi: 10.1080/10669817.2023.2281650 PMC11216239 38146749

[19] Hayton T, Gross A, Basson A, et al. Psychometric properties of clinician-reported and performance-based outcomes cited in a scoping review on spinal manipulation and mobilisation for paediatric populations with diverse medical conditions: a systematic review. J Manual Manipulative Ther. 2023;1–29. doi: 10.1080/10669817.2023.2269038 PMC11216262 38070150

[20] Hong QN, Pluye P, Fàbregues S, et al. The Mixed Methods Appraisal Tool (MMAT) version 2018 for information professionals and researchers. Education Info. 2018 Jan 1;34(4).285–91.

[21] National Health and Medical Research Council. NHMRC additional levels of evidence and grades for recommendations for developers of guidelines- stage 2 consultation. 2009. www.nhmrc.gov.au/publications/synopses/cp65syn.htm

[22] Joanna Briggs Institute. JBI levels of evidence 2013. [cited 2023 Oct 27]. Available from: https://jbi.global/sites/default/fles/2019-05/JBI-Levels-of-evidence_2014_0.pdf

[23] Prinsen CAC, Mokkink LB, Bouter LM, et al. COSMIN guideline for systematic reviews of patient-reported outcome measures. Qual Life Res. 2018;27(5):1147–57. doi: 10.1007/s11136-018-1798-3 29435801 PMC5891568

[24] Dice JL, Brismee J-M, Froment FP, et al. Spinal manipulation and mobilisation among infants, children, and adolescents: an international Delphi survey of expert physiotherapists. J Manual Manipulative Ther. 2024;1–11. doi:10.1080/10669817.2024.2327782. PMC11216234 38484120

[25] Snilstveit B, Vojtkova M, Bhavsar A, et al. Evidence & gap maps: a tool for promoting evidence informed policy and strategic research agendas. J Clinical Epidemiol. 2016;79:120–29. doi: 10.1016/j.jclinepi.2016.05.015 27387966

[26] Schuller-Martínez B, Meza N, Pérez-Bracchiglione J, et al. Graphical representation of the body of the evidence: the essentials for understanding the evidence gap map approach. Medwave. 2021;21(3):e8164–e8164. doi: 10.5867/medwave.2021.03.8164 34081682

[27] Abu-Arafeh I, Hershey AD, Diener H-C, et al. Guidelines update: guidelines of the international headache society for controlled trials of preventive treatment of migraine in children and adolescents, 1st edition – an experience-based update. Cephalalgia. 2023;43(5):03331024231178239. doi: 10.1177/03331024231178239 37226450

[28] (IHS) Olesen J. Headache classification committee of the International headache society (IHS) the international classification of headache disorders, 3rd edition. Cephalalgia. 2018;38(1):1–211. doi: 10.1177/0333102417738202 29368949

[29] Rowe DE, Feise RJ, Crowther ER, et al. Chiropractic manipulation in adolescent idiopathic scoliosis: a pilot study. Chiropr Man Therap. 2006;14(1):15. 10.1186/1746-1340-14-15 PMC1560145 16923185

[30] Evans R, Haas M, Schulz C, et al. Spinal manipulation and exercise for low back pain in adolescents: a randomized trial. Pain. 2018;159(7):1297–307. doi: 10.1097/j.pain.0000000000001211 29596158 PMC6205160

[31] L’Ecuyer JL. Congenital occipitalization of the atlas with chiropractic manipulations: a case report. Nebr State Med J. 1959;44:546–550. 14413090

[32] Ziv I, Rang M, Hoffman HJ. Paraplegia in osteogenesis imperfecta. A case report. J Bone Joint Surg Br. 1983;65-B(2):184–185. doi: 10.1302/0301-620X.65B2.6826628 6826628

[33] Jacobi G, Riepert T, Kieslich M, et al. Über einen Todesfall während der Physiotherapie nach Vojta bei einem 3 Monate alten Säugling - Fallbeschreibung und Bemerkungen zur Manualtherapie bei Kindern -. Klin Padiatr. 2001;213(2):76–85. 11305197 10.1055/s-2001-12881

[34] Shafrir Y, Kaufman BA. Quadriplegia after chiropractic manipulation in an infant with congenital torticollis caused by a spinal cord astrocytoma. J Paediatr. 1992;120(2):266–269. doi: 10.1016/s0022-3476(05)80440-8 1735825

[35] Borusiak P, Biedermann H, Bosserhoff S, et al. Lack of efficacy of manual therapy in children and adolescents with suspected cervicogenic headache: results of a prospective, randomized, placebo-controlled, and blinded trial. Headache. 2010;50(2):224–30. doi: 10.1111/j.1526-4610.2009.01550.x 19845788

[36] Zimmerman AW, Kumar AJ, Gadoth N, et al. Traumatic vertebrobasilar occlusive disease in childhood. Neurology. 1978;28(2):185–8. doi: 10.1212/wnl.28.2.185 563999

[37] Saedt E, Driehuis F, Hoogeboom TJ, et al. Common manual therapy practices in the Netherlands for infants with upper cervical dysfunction: a prospective cohort study. J Manipulative Physiol Ther. 2018;41(1):52–61. 29254625 10.1016/j.jmpt.2017.08.003

[38] Miller J, Newell D. Prognostic significance of subgroup classification for infant patients with crying disorders: a prospective cohort study. J Can Chiropractic Assoc. 2012;56(1):40–48. PMC3280117 22457540

[39] Koch LE, Biedermann H, Saternus KS. High cervical stress and apnoea. Forensic science international. Forensic Sci Int. 1998;97(1):1–9. doi: 10.1016/S0379-0738(98)00124-8 9854836

[40] Wilson PM, Greiner MV, Duma EM. Posterior rib fractures in a young infant who received chiropractic care. Pediatrics. 2012;130(5):e1359–62. doi: 10.1542/peds.2012-0372 23027167

[41] Leboeuf C, Brown P, Herman A, et al. Chiropractic care of children with nocturnal enuresis: a prospective outcome study. J Manipulative Physiol Ther. 1991;14(2):110–115. 2019820

[42] Sawyer CE, Evans RL, Boline PD, et al. A feasibility study of chiropractic spinal manipulation versus sham spinal manipulation for chronic otitis media with effusion in children. J Manipulative Physiol Ther. 1999;22(5):292–298. 10395431 10.1016/s0161-4754(99)70061-8

[43] Bellman M, Byrne O, Sege R. Developmental assessment of children. Br Med J. 2013;346(2):e8687–e8687. doi: 10.1136/bmj.e8687 23321410

[44] Marwaha S, Goswami M, Vashist B. Prevalence of principles of Piaget’s theory among 4-7-year-old children and their correlation with IQ. J Clin Diagn Res. 2017;11(8):ZC111. doi: 10.7860/JCDR/2017/28435.10513 28969287 PMC5620909

[45] Negrini S, Donzelli S, Aulisa AG, et al. SOSORT guidelines: orthopaedic and rehabilitation treatment of idiopathic scoliosis during growth. Scoliosis Spinal Disord. 2016;13(1):3. doi: 10.1186/s13013-017-0145-8 published Online First: 2018/02/13 PMC5795289 29435499

[46] Kaplan SL, Coulter C, Sargent B. Physical therapy management of congenital muscular torticollis: a 2018 evidence-based clinical practice guideline from the American physical therapy association academy of paediatric physical therapy. Pediatr Phys Ther Am Phys Ther Assoc. 2018;30(4):240. doi: 10.1097/PEP.0000000000000544 PMC8568067 30277962

[47] Picart T, Beuriat P, Szathmari A, et al. Positional cranial deformation in children: a plea for the efficacy of the cranial helmet in children. Neurochirurgie. 2020;66(2):102–09. doi: 10.1016/j.neuchi.2019.10.011 31958410

[48] Milne N, Cristofoli T, Stiel K, et al. Review protocol: effectiveness of spinal manipulation and mobilisation for treating headache, and neck and back pain in paediaric populations a systematic review. Charlottesville, VA: Center for Open Science Research; 2023.

[49] Verhoef M. Survey of Canadian chiropractor’s involvement in the treatments of patients under the age of 18. J Can Chiropractic Assoc. 1999;43:50–57.

[50] Castilla A, Gonzalez M, Kysh L, et al. Informing the physical therapy management of congenital muscular torticollis clinical practice guideline: a systematic review. Pediatr Phys Ther. 2023;35(2):190–200. doi: 10.1097/PEP.0000000000000993 36637442

[51] Mawji A, Vollman AR, Hatfield J, et al. The incidence of positional plagiocephaly: a cohort study. Pediatrics. 2013;132(2):298–304. doi: 10.1542/peds.2012-3438 23837184

[52] Peitsch WK, Keefer CH, LaBrie RA, et al. Incidence of cranial asymmetry in healthy newborns. Pediatrics. 2002;110(6):e72–e72. doi: 10.1542/peds.110.6.e72 12456939

[53] Biedermann H. Manual therapy in children. London: Churchill Livingstone; 2004.

[54] Happle C, Wetzke M, Hermann EJ, et al. “Cases against KiSS”: Ein diagnostischer Algorithmus des frühkindlichen Torticollis. Klinische Pädiatrie. 2009;221(7):430–435. 20013566 10.1055/s-0029-1243162

[55] Aarnivala H, Vuollo V, Harila V, et al. The course of positional cranial deformation from 3 to 12 months of age and associated risk factors: a follow-up with 3D imaging. Eur J Pediatr. 2016;175(12):1893–1903. 27624627 10.1007/s00431-016-2773-z

[56] Olesen J. Headache classification committee of the international headache society (IHS) the international classification of headache disorders. Cephalalgia. 2018;38(1):1–211. 10.1177/0333102417738202 29368949

[57] Zorzela L, Loke YK, Ioannidis JP, et al. PRISMA harms checklist: improving harms reporting in systematic reviews. Br Med J. 2016;352. doi: 10.1136/bmj.i157 26830668

[58] Huhn K, Gilliland SJ, Black LL, et al. Clinical reasoning in physical therapy: a concept analysis. Phys Ther. 2019;99(4):440–56. doi: 10.1093/ptj/pzy148 30496522

[59] Doody C, McAteer M. Clinical reasoning of expert and novice physiotherapists in an outpatient orthopaedic setting. Physiotherapy. 2002;88(5):258–68. doi: 10.1016/S0031-9406(05)61417-4

[60] Jensen GM, Gwyer J, Shepard KF. Expert practice in physical therapy. Phys Ther. 2000;80(1):28–43. doi: 10.1093/ptj/80.1.28 discussion 44-52. 10623958

[61] Posadzki P, Kyaw BM, Dziedzic A, et al. Osteopathic manipulative treatment for paediatric conditions: an update of systematic review and meta-analysis. J Clin Med. 2022;11(15):4455. doi: 10.3390/jcm11154455 35956072 PMC9369972

[62] Keating G, Hawk C, Amorin-Woods L, et al. Clinical practice guideline for best practice management of paediatric patients by chiropractors: results of a Delphi consensus process. J Integ Complement Med. 2023. doi:10.1089/jicm.2023.0010. PMC10954607 37902954

[63] Blanpied PR, Gross AR, Elliott JM, et al. Neck pain: revision 2017. J Orthop Sports Phys Ther. 2017;47(7):A1–A83. doi: 10.2519/jospt.2017.0302 28666405

[64] Delitto A, George SZ, Van Dillen L, et al. Low back pain: clinical practice guidelines linked to the international classification of functioning, disability, and health from the orthopaedic section of the American physical therapy association. J Orthop Sports Phys Ther. 2012;42(4):A1–A57. doi: 10.2519/jospt.2012.42.4.A1 18758050

[65] Childs JD, Cleland JA, Elliott JM, et al. Neck pain: clinical practice guidelines linked to the international classification of functioning, disability, and health from the orthopedic section of the American physical therapy association. J Orthop Sports Phys Ther. 2008;38(9):A1–A34. doi: 10.2519/jospt.2008.0303 18758050

[66] George SZ, Fritz JM, Silfies SP, et al. Interventions for the management of acute and chronic low back pain: revision 2021. J Orthop Sports Phys Ther. 2021;51(11):CPG1–CPG60. doi: 10.2519/jospt.2021.0304 PMC10508241 34719942

[67] Maher CG, Archambeau A, Buchbinder R, et al. Introducing Australia’s clinical care standard for low back pain. J Med Imag Rad Onc. 2023;67(4):404–408. 10.1111/1754-9485.13532 37125441

